# Differentiation-Dependent Interpentameric Disulfide Bond Stabilizes Native Human Papillomavirus Type 16

**DOI:** 10.1371/journal.pone.0022427

**Published:** 2011-07-19

**Authors:** Michael J. Conway, Linda Cruz, Samina Alam, Neil D. Christensen, Craig Meyers

**Affiliations:** 1 Department of Microbiology and Immunology, The Pennsylvania State University College of Medicine, Hershey, Pennsylvania, United States of America; 2 Department of Pathology, The Pennsylvania State University College of Medicine, Hershey, Pennsylvania, United States of America; National Institute of Health - National Cancer Institute, United States of America

## Abstract

Genetic and biochemical analyses of human papillomavirus type 16 (HPV16) capsids have shown that certain conserved L1 cysteine residues are critical for capsid assembly, integrity, and maturation. Since previous studies utilized HPV capsids produced in monolayer culture-based protein expression systems, the ascribed roles for these cysteine residues were not placed in the temporal context of the natural host environment for HPV, stratifying and differentiating human tissue. Here we extend upon previous observation, that HPV16 capsids mature and become stabilized over time (10-day to 20-day) in a naturally occurring tissue-spanning redox gradient, by identifying temporal roles for individual L1 cysteine residues. Specifically, the C175S substitution severely undermined wild-type titers of the virus within both 10 and 20-day tissue, while C428S, C185S, and C175,185S substitutions severely undermined wild-type titers only within 20-day tissue. All mutations led to 20-day virions that were less stable than wild-type and failed to form L1 multimers via nonreducing SDS-PAGE. Furthermore, Optiprep-fractionated 20-day C428S, C175S, and C175,185S capsids appeared permeable to endonucleases in comparison to wild-type and C185S capsids. Exposure to an oxidizing environment failed to enhance infectious titers of any of the cysteine mutants over time as with wild-type. Introduction of these cys mutants results in failure of the virus to mature.

## Introduction

Human papillomaviruses (HPVs) contain a single, circular dsDNA genome of approximately 8 kb which associates with cellular histones to form a minichromosome. This minichromosome is encapsidated within a nonenveloped, icosahedral capsid composed of the late proteins, L1 and L2. Similar to polyomaviruses, 360 monomers of the major capsid protein L1 assemble into 72 pentameric capsomeres which are geometrically arranged on a T = 7 icosahedral lattice [1], [2], [3], [4], [5]. Recent cryo-electron microscopy image reconstructions of HPV16 pseudovirions (PsV) support previous biochemical analyses suggesting that capsids can be maximally occupied with 72 L2 proteins per virion [6], [7].

Due to the relative inefficiency of organotypic culture in the production of native HPVs, structural analyses of HPV capsids has been largely limited to the utilization of more efficient monolayer culture-derived papillomavirus particles such as virus-like particles (VLPs), and PsV [6], [7], [8], [9], [10], [11], [12], [13], [14], [15], [16], [17], [18], [19], [20]. X-ray crystallographic data has been obtained from an HPV16 L1 with N-terminal and C-terminal deletions forming a 12-pentamer, small VLP [13]. However, the small size of this structure and lack of disulfide bonds leave many of the structural details of native HPV16 open for interpretation [13], [14], [15], [16], [17], [18], [19], [20]. Recently, a high-resolution cryo-electron microscopy image reconstruction of native bovine papillomavirus particles (BPV) depicted two separate disulfide bonds. The C171-C426 disulfide bond forms an interpentameric interaction between two highly conserved cysteine residues homologous to C175 and C428 in HPV16. The BPV C22–C473 disulfide bond forms an intrapentameric interaction between two less conserved cysteine residues that are only conserved among papillomaviruses from ruminants [21]. Supporting the presence of a C171–C426 disulfide bond in other HPV types, mass spectrometry analysis of L1 dimers from non-reduced HPV18 VLPs depicted the presence of a C175–C429 disulfide bond [14]. Genetic and biochemical analyses of HPV16 and HPV33 VLPs and PsV have also shown the importance of these conserved cysteines in the structural integrity of particles [6], [14], [16], [18], [20]. Previous studies have also highlighted the potential structural role of C185, which neighbors C175 on the EF loop of HPV16 L1. C185 has been shown to affect the structural integrity of HPV16 VLPs and has been theorized to play a role in the production of aberrant L1 dimers [6], [16].

Since previous studies into the disulfide bonding patterns of HPV16 capsids utilized particles produced in monolayer culture, or from fully developed BPV-containing warts, disulfide interactions were not placed in the temporal context of stratified and differentiating human tissue. Here we extend upon our previous observation, that HPV16 capsids become stabilized over time [22], [23], [24]. Our previous studies showed that HPV16 formed infectious but unstable, poorly antibody-mediated neutralizable particles after 10 days of growth in host tissue, requiring another ten days of growth to form stable, antibody-mediated neutralizable particles. By utilizing a tissue-spanning redox gradient, we identified temporal roles for individual C175, C185, and C428 L1 cysteine residues. Only the C175S substitution severely undermined wild-type levels of infectious virus within both 10- (reducing environment) and 20-day (oxidative environment) tissue, while C428S, C185S, and C175,185S substitutions all undermined wild-type levels of infectious virus only within 20-day tissue suggesting they are important to stabilize the particles over time. Interestingly, upon fractionation more noninfectious defective virus appeared to co-migrate with infectious 20-day C428S, C175S, and C175,185S virions in comparison to wild-type and C185S virions, suggesting that the mutations permeabilized the capsid lumen to treatment with an endonuclease. In addition, treatment of raft cultures with oxidized glutathione (GSSG) failed to enhance infectious titers within any mutant tissues compared to wild-type, suggesting that these residues are important in the redox-dependent enhancement observed of wild-type HPV16 infectivity in a more oxidative environment. Together these results allowed us to hypothesize that a complex, temporal interplay of disulfide bond formation, disruption, and reformation occurs between C175, C185, and C428. These data support previous reports utilizing VLPs or PsVs that an initial C185–C428 disulfide bond may form in order to prime formation of a C175–C428 disulfide bond during capsid assembly in stratifying and differentiating human epithelial tissue [6], [16].

## Results

### Establishment of stable cell lines maintaining episomal HPV16 DNA

**Figure 1:**
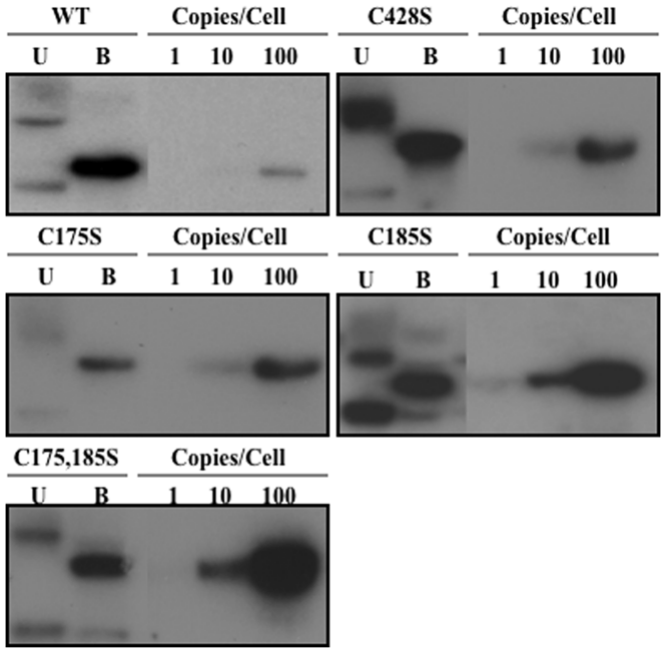
Episomal maintenance of wild-type and mutant viral genomes. Southern blot hybridization of a representative HPV16(114/B)-infected HFK cell lines containing wild-type (WT), and C428S, C175S, C185S, and C175,185S mutant genomes. Uncut (U) and single *BamHI* cut (B) episomal genomes are shown in addition to 1, 10, and 100 viral genome copies per 5 µg of total genomic DNA.

**Figure 2:**
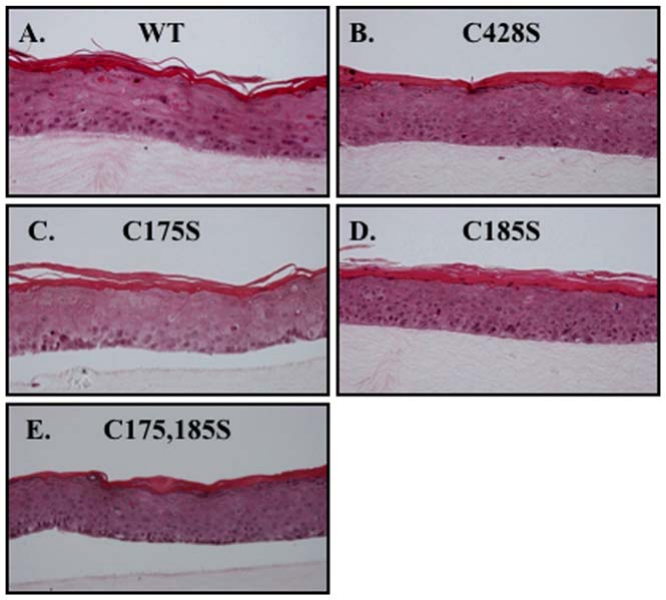
Stratification of wild-type and mutant organotypic cultures. Hematoxylin and eosin staining of paraffin-embedded, 10-day wild-type (WT), C428S, C175S, C185S, and C175,185S-infected organotypic tissue.

**Figure 3:**
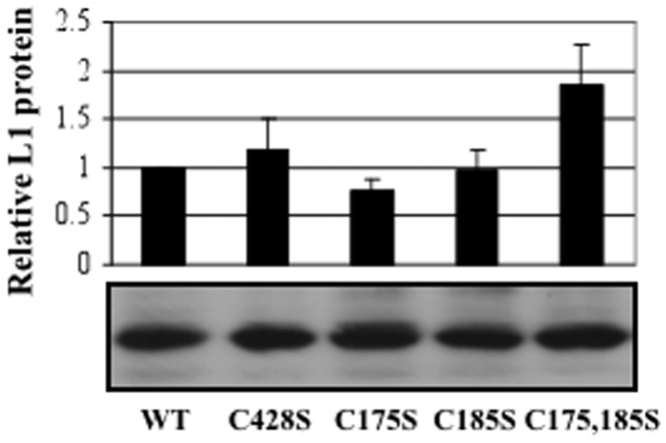
Expression of L1 in wild-type and mutant 10 day tissues. Western blot analyses of equal volumes from WT, C428, C175, C185S, and C175,185S 10-day virus preparations. A representative Western blot is shown below while a graph depicting the relative L1 protein expression per viral preparation via densitometry is shown above. The results are means and standard deviations from three independent experiments.

**Figure 4:**
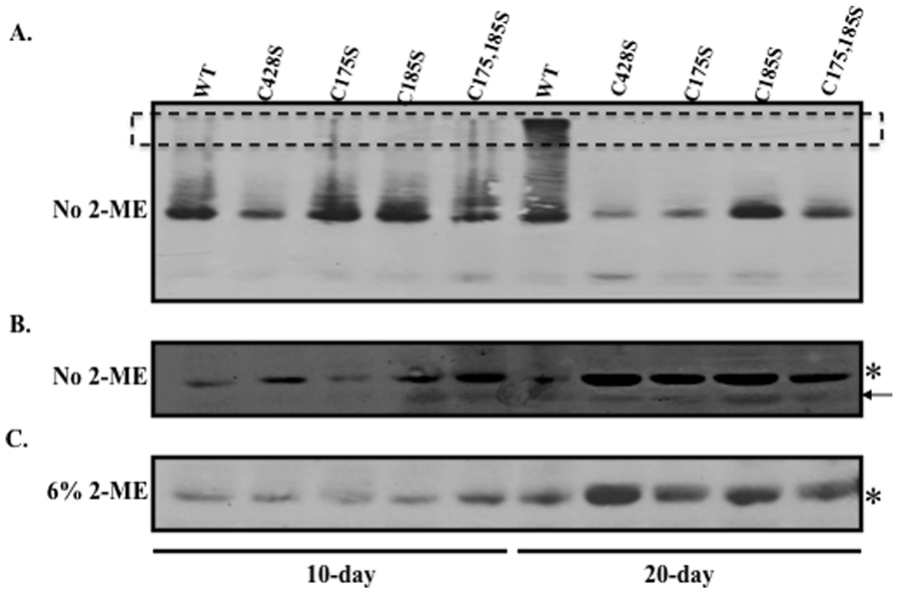
Nonreducing Western blot analyses of 10 and 20-day wild-type and mutant viruses. Representative Western blot images of equal volumes of 10 and 20-day wild-type and mutant viruses run under nonreducing (A–B) and reducing (C) conditions. Monomeric L1 (lower band in A) under nonreducing conditions is shown overexposed in (B). Monoclonal H16.J4 was used to detect HPV16 L1. Multimeric L1 species are indicated by a box. An arrow points to a faster migrating L1 species which has been previously shown to be present in greater concentration within purified virions [23].

To develop producer cell lines which can synthesize organotypic culture-derived native virions from differentiating epithelia, primary human foreskin keratinocytes (HFKs) were electroporated with linearized wild-type and site-directed mutagenized HPV16(114/B) DNA [9]. The recircularization and maintenance of episomal HPV16 viral genomes for representative HPV16 L1 C428S, C175S, C185S, and C175,185S cell lines can be seen in Figure 1. Data for wild-type and C428S HPV16-containing cell lines have been published previously [22], [25]. The total number of episomal copies per cell was less in mutant cell lines (∼50–200 copies/cell) than the wild-type cell line utilized (>1,000 copies/cell). Importantly, the lower copy numbers observed in monolayer culture in the mutant cell lines are not significant regarding productivity of tissues since previous reports have suggested that copy number does not directly correlate with the titer of HPV-infected organotypic tissues [25], [26], [27]. Quantifying genome equivalents and the detection of the major capsid protein via Western blot allowed for the analysis of the relative infectivity of viral stocks produced by HPV-infected organotypic tissues. For each mutant genome, multiple episomal DNA-containing cell lines were produced and utilized in experiments. In addition, L1 ORFs were sequenced to verify the existence of the intended mutation, and the absence of erroneous mutations. Only then were stable cell lines allowed to grow as stratified and differentiated epithelial tissues in organotypic culture (Fig. 2). Importantly, mutant-infected tissues did not show a significantly different phenotype than wild-type-infected tissues (Fig. 2) and total L1 protein concentration was similar in wild-type compared to mutant in 10-day tissues (Fig. 3A and 4C).

### L1 multimer formation in 10- and 20-day wild-type and mutant virions

Previous literature suggests that HPV16 and BPV1 PsV can mature within 293TT cell lysates if incubated overnight at 37°C [6]. This maturation event is characterized by the formation of higher-ordered L1 multimers, presumably formed through interpentameric disulfide bonds [6]. Similarly, we have previously shown that native HPV16 virions are stabilized by a tissue-spanning redox gradient within human epithelial tissue. Here we hypothesized that this stabilization event was mediated by the conserved cysteine residues C175 and C428 which have been predicted to form a critical interpentameric disulfide bond in numerous biochemical and genetic analyses [6], [14], [16], [18], [21]. To test this hypothesis, nonreducing Western blot analyses of 10- and 20-day wild-type and mutant viral preparations were performed (Fig. 4A–C). Consistent with our hypothesis that 10-day virions lack a stabilizing interpentameric disulfide bond, only monomeric L1 was observed in both 10-day wild-type and mutant viruses (Fig. 4A–B). Consistent with our hypothesis that C175 and C428 are involved in stabilizing the particles over time, under nonreducing conditions L1 multimer formation was only observed in wild-type 20-day virus (Fig. 4A). Since a significant increase in L1 protein concentration was observed in wild-type and mutant 20-day tissues compared to 10-day tissues, nonreducing Western blots were overexposed to determine if multimers were detected in 10-day virus. No multimers were observed in wild-type or mutant 10-day virus in overexposed nonreducing Western blots (data not shown). L1 multimer formation in wild-type 20-day virus was always accompanied by an equivalent loss in the monomeric population (Fig. 4A–C). The lack of L1 multimer formation in all 20-day mutant viruses supports a role for, not only C175 and C428 in a stabilizing interpentameric disulfide bond, but also for C185. The total amount of L1 protein in the various tissues that was able to run on an SDS-PAGE gel is shown in figure 4C. Intact wild-type virions likely fail to enter the gel as easily as collapsed C428S virions resulting in more L1 protein signal. In our experience native virions are not completely reduced to monomers in the presence of 2-ME and SDS.

### DNA encapsidation efficiency of wild-type and mutant virions

Previous studies indicate that C428S, C175S, and C185S substitutions in HPV16 VLPs and PsV dramatically alters either initial assembly of viral particles, or their stabilities after purification [6], [16]. To quantitatively assess capsid stability, the efficacy of wild-type versus mutant L1 proteins to assemble and protect viral genomes in the context of stratified and differentiated epithelial tissues was determined. This was performed by growing rafts for 10 and 20 days. Viral stocks were either treated with benzonase in order to eliminate exogenous viral genomes present within the lysate leaving only endonuclease-resistant or encapsidated genomes, or left untreated so that total viral genomes present could be quantified [22], [23]. Benzonase treatment has been previously reported to eliminate both chromatin-associated and chromatin-non-associated DNA, in addition to a large population of endonuclease-susceptible viral genomes, while leaving the population of protected viral genomes for downstream analysis [22], [23]. Analysis of non-benzonase-treated viral stocks for total viral DNA showed no statistical difference between wild-type and mutant samples (data not shown). While total viral genomes remained statistically similar in these viral stocks, mutations in the viral genome and host keratinocyte genetic background could alter translation efficiencies of L1 transcripts. Because of this, total L1 protein within 10-day tissues was estimated by Western blot analysis (Fig. 3). While a slight increase in total L1 protein levels was observed in 10-day C175,185S virus compared to wild-type, no statistical difference was observed for 10-day C428S, C175S, or C185S viruses. These results suggested that mutations and/or donor tissues had no major effect on L1 protein expression levels.

**Figure 5:**
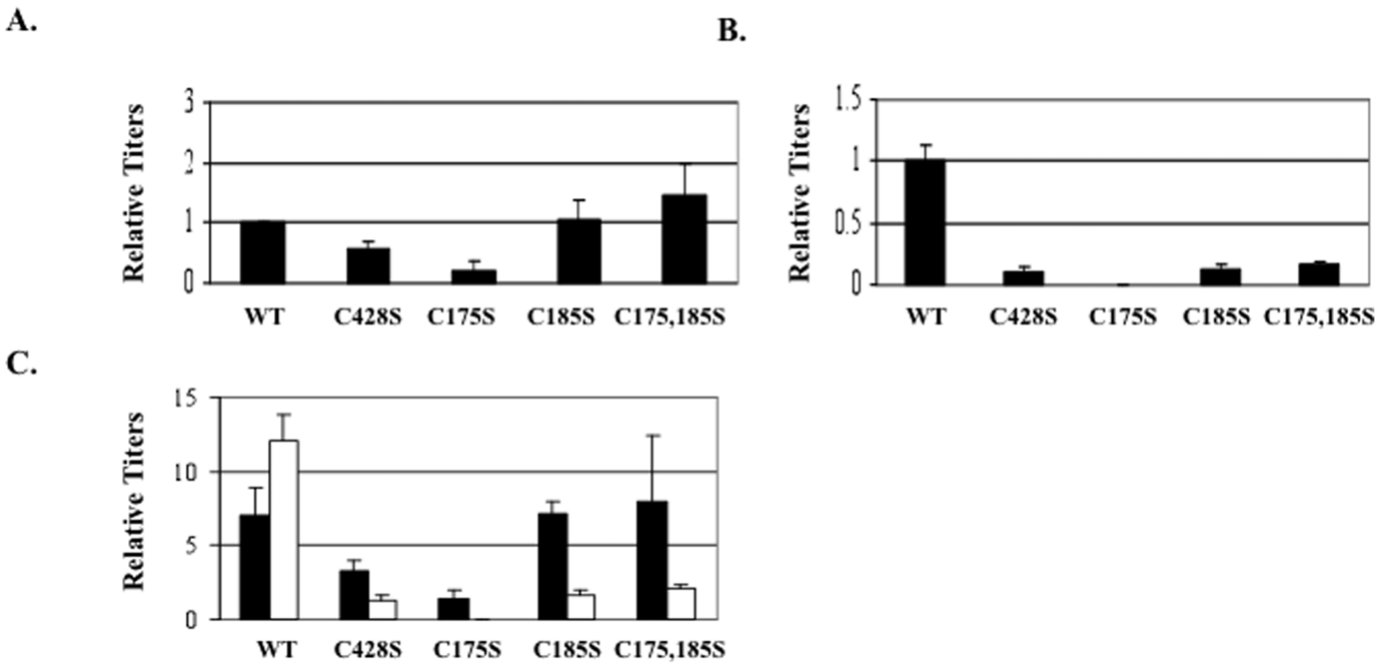
Titers in 10 and 20-day wild-type and mutant viruses. Titers were measured using a SYBR-green based genome encapsidation assay. (A) SYBR green-based DNA encapsidation assay relative quantification of 10-day wild-type (WT), C428S, C175S, C185S, and C175,185S viruses. WT is set to 1.0. (B) SYBR green-based DNA encapsidation assay relative quantification of 20-day wild-type (WT), C428S, C175S, C185S, and C175,185S viruses. WT is set to 1.0. (C) Side-by-side comparison of 10 (black bars) and 20-day (white bars) raw data from A and B. Relative y-axis values represent copy number controls used in the standard curve. The results are means and standard errors for at least three independent experiments.

HPV titers are measured similar to other viruses such as the Kaposi's sarcoma virus (KSHV), quantifying viral genome equivalents [28], [29], [30], [31], [32]. In Fig. 5A, the relative amounts of HPV titers in 10-day viral preparations are shown. Ten-day C428S, C175S, C185S, and C175,185S mutants had titers that were 54%±14, 20%±15, 110%±30, and 145%±51, respectively, of wild-type titers (Table 1). This suggests that C428 was mildly and C175 was largely important for early viral genome encapsidation. The observation that 10-day C428S viruses had titers that were 54% of 10-day wild-type virus was surprising since the C428S mutation previously led to complete capsid disassembly when integrated into HPV16 and HPV33 VLPs, and HPV16 PsV [6], [16], [18]. We theorize that proper covalent bonding prevents exposure of viral genomes to benzonase.

**Table 1:** Titers and infectivity of cys mutants.

	Titers (% change compared to wild-type ± SD)		Infectivity (% change compared to wild-type ± SD)		Infectivity Relative To L1 Protein (% change compared to wild-type ± SD)	
Mutants	10-day	20-day	10-day	20-day	10-day	20-day
C428S	54%±14	8%±4	86%±24	64%±15	41%±28	5%±3
C175S	20%±15	1%±0.05	3.5%±1.2	46%±8	0.3%±0.2	0.1%±0.1
C185S	110%±30	13%±4	87%±7	152%±10	148%±60	20%±3
C175,185S	145%±51	14%±6	38%±7	31%±4	49%±26	10%±9

Fig. 5B shows the titers measured from 20-day viral preparations. Compared to 20-day wild-type titers, 20-day C428S, C175S, C185S, and C175,185S virus preparations contained titers of 8%±4, 1%±0.05, 13%±4, and 14%±6, respectively. In addition, side-by-side analysis of 10-day and 20-day tissues (Fig. 5A–B) shows that from 10 to 20 days, the titers increased in wild-type-infected tissues, while the titers dropped in all mutant-infected tissues (Fig. 5C). This dramatic decrease in titers occurred while total L1 protein concentration in 20-day virus preparations appeared equal or higher in mutant preparations compared to wild-type virus preparations (Fig. 4C). Such dramatic decreases in titers apparent in 20-day virus preparations compared to 20-day wild-type virus preparations supports that, over time, C428S, C175S, and C185S facilitate necessary steps in virion maturation. This suggests that in the oxidative environment of tissue, disulfide bonds are necessary to maintain a stable particle.

### Infectivity of wild-type and mutant virions

**Figure 6:**
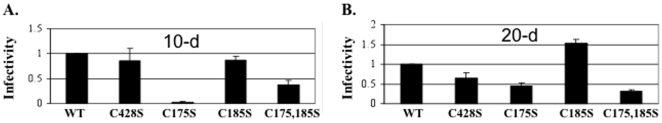
Infectivity of 10 and 20-day wild-type and mutant virions. (A) Infectivity was assayed using equal titers of 10-day wild-type (WT), C428S, C175S, C185S, C175,185S viruses. Relative endonuclease-resistant genome data in Fig. 5A was used to normalize relative infectivity data in A. WT is set to 1.0. (B) Infectivity of 20-day wild-type (WT), C428S, C175S, C185S, C175,185S viruses. WT is set to 1.0.The results are means and standard errors for at least three independent experiments.

While significant reductions in titers were detected in 10 and 20-day mutant virus preparations, all preparations contained quantifiable titers (Fig. 5A–C). These results demonstrated that, while inefficient, all of the cys mutant L1 proteins tested can encapsidate and protect viral genomes from endonucleases. To determine infectivity of the mutant particles, a previously reported RT-qPCR-based infectivity assay measuring the abundance of E1∧E4 early transcript was performed [22], [23]. Infectivity of mutant particles was established on equal multiplicity of infection (MOI) based on the titers measured or by total L1 protein. Infectivity of 10-day C428S, C175S, C185S, and C175,185S were 86%±24, 3.5%±1.2, 87%±7, and 38%±7, respectively, of wild-type (Fig. 6) (Table 1). The drop in infectivity observed with C175S and C175,185S does not appear to be due to the lack of an interpentameric disulfide bond with C428 since C428S mutant virus were nearly as infectious as wild-type (Fig. 6). We next tested infectivity of 20-day C428S, C175S, C185S, and C175,185S virus stocks. When compared to wild-type, the infectivity for 20-day C428S, C175S, C185S, and C175,185S was 64%±15, 46%±8, 152%±10, and 31%±4, respectively (Table 1).

When we compared to 10-day wild-type virus, the infectivity relative to L1 protein of 10-day C428S, C175S, C185S, and C175,185S viruses was 41%±28, 0.3%±0.2, 148%±60, and 49%±26, respectively. Compared to 20-day wild-type, the infectivityrelative to L1 protein of 20-day C428S, C175S, C185S, and C175,185S viruses was 5%±3, 0.1%±0.1, 20%±3, and 10%±9, respectively.

### Capsid stabilities of wild-type and mutant virions

In Fig. 5A–C, genome encapsidation of viral genomes was dramatically reduced in 10-day C175S viruses in addition to 20-day C428S, C175S, C185S, and C175,185S viruses as compared to wild-type. Rather than resulting from structural defects in capsid assembly, reduction in endonuclease resistant genomes might be due to a reduced ability of mutant viral genomes to synthesize L1. Western blot analyses of wild-type and mutant virus infected tissue showed that L1 expression of the mutant viruses was similar to or greated than that of wild-type (Fig. 3B and Fig. 4C). Considering the previously ascribed structural roles for C428, C175, and C185 and that no decrease in L1 expression was observed in tissues, we hypothesized that the reductions in endonucease-resistant genomes observed in Fig. 5A–C was due to structural defects in the mutant capsids.

**Figure 7:**
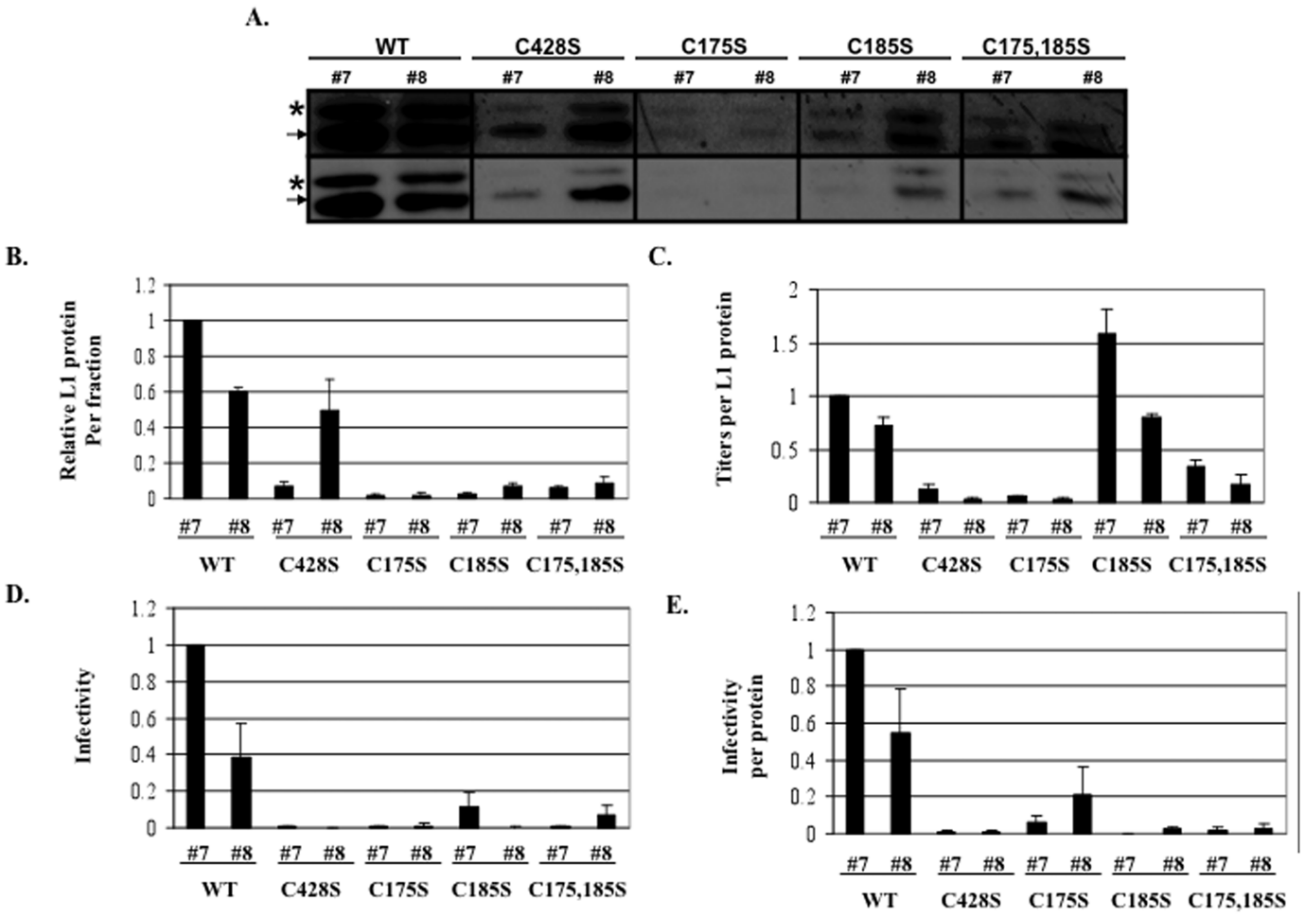
Analysis of infectious Optiprep fractions. (A) Western blot analysis of L1 in highly infectious Optiprep fractions #7 and #8 from Optiprep-fractionated 20-day wild-type (WT), C428S, C175S, C185S, and C175,185S viruses. The two rows are replicates with the top being overexposed. Higher and lower molecular weight species of L1 are indicated. (B) Densitometric analysis of the faster migrating L1 species (i.e. 55 kD) in three independent Western blots as shown in A. WT fraction #7 was set to 1.0. (C) Titers per fraction, normalized to the relative amount of L1 protein within fractions. WT fraction #7 was set to 1.0. (D) Infectivity of 20-day wild-type (WT), C428S, C175S, C185S, C175,185S virions within fractions #7 and #8. WT is set to 1.0. (E) Infectivity of 20-day wild-type (WT), C428S, C175S, C185S, C175,185S virions within fractions #7 and #8. Relative L1 protein content per fraction were used to normalize relative infectivity data within fractions. WT is set to 1.0.The results are means and standard errors for at least three independent experiments. The arrow points to the L1 band with the same motility as seen with L1 from PsV [23]. The asterisk marks the slow mobility L1 band seen only in native HPV16 virion [23].

We have previously demonstrated that highly infectious wild-type virus migrates into Optiprep fractions #7 and #8 [22], [23]. Therefore, to further examine the stability of these mutants, 20-day viruses were Optiprep-fractionated and Western blot analyses of L1 were performed on mature virus-containing, fractions #7 and #8 (Fig. 7A–B). It was observed that more L1 protein was found in fractions #7 and #8 of wild-type compared to mutant preparations (Fig. 7A–B). This decrease in L1 protein in mutant fractions occurred while total L1 protein concentration in 20-day viruses appeared equal or higher in mutant viruses compared to wild-type virus (Fig. 4C). We hypothesized that, if mutant capsids were more unstable than wild-type capsids, the majority of mutant L1 protein would migrate to the top fractions of the Optiprep gradient due to the destruction of capsids because of the high forces of ultracentrifugation. Previously published data in our laboratory suggested that such capsid destruction is prevalent in immature, 10-day wild-type HPV16 virions and is mostly absent in the more stable HPV16 20-day wild-type virions [23].

**Figure 8:**
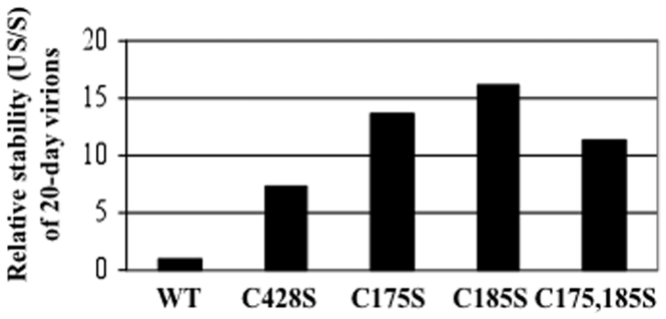
Relative stabilities of 20-day wild-type versus mutant virions. 20-day wild-type (WT), C428S, C175S, C185S, and C175,185S viruses were Optiprep-fractionated. Fractions were assayed by a SYBR green-based DNA encapsidation assay to detect total endonuclease-resistant genomes per fraction. As a measure of capsid stability, the sum of genomes in the uppermost gradient fractions (fractions #1 through #4) was described as “unstable” (US), which were genomes either not associated or disassociated from an intact capsid during ultracentrifugation. The sum of genomes flanking highly infectious fractions (fractions #6 through #9) was described as “stable” (S) since they were associated with intact capsids. Relative capsid stability is described by the ratio US/S, whereby higher values indicate more unstable virions [23].

The instability of mutant capsids was compared to wild-type capsids by assessing the relative stability of virus within the Optiprep gradient (Fig. 8). The relative stability of virus was measured by the ratio of unstably encapsidated viral genomes (sum of fractions #1 through #4) over stably encapsidated viral genomes (sum of fractions #6 through #9) [23]. We have shown previously that 10-day wild-type HPV16 virions have a stability ratio of 26.0, while their 20-day counterparts have a stability ratio of 1.0, suggesting that HPV16 virions are stabilized over time [23]. While 20-day wild-type virions had a relative stability of 1.0, 20-day C428S, C175, C185S, and C175,185S virions had relative stabilities of 7.3, 13.8, 16.1, and 11.4, respectively, suggesting that these mutations rendered capsids less stable than 20-day wild-type virions (Fig. 8).

To determine if Optiprep-fractionated mutant virions retained full infectivity, we performed the RT-qPCR-based infectivity assay on each Optiprep fraction (Fig. 7D–E). Using equal titers (Fig. 7D) or normalizing using total protein concentration (Fig. 7E, it was apparent that, in contrast to the infectivity of mutant virions directly from 20-day viral preparations (Fig. 6D), the infectivity of Optiprep-fractionated mutant virions was dramatically less than wild-type virions. This result indicated that mutant virions that did migrate into into infectious fractions did not retain the same level of infectivity as their counterparts tested directly from unfractionated viral preparations. Gradient-purified mutant virions might not be as stable as wild-type virions and the observed reduction in total L1 protein concentrations within mutant Optiprep gractions might by due to the instability of their capsids. Optiprep fractions outside of fractions #7 and #8 were scanned but yielded no higher infectivity peaks. This suggested that the result was not due to a shift in the migration of mutant capsids, but rather that the capsids were falling apart, or that these residues were important for an unknown entry step.

Interestingly, when we compared the total amount of endonuclease-resistant genomes within each Optiprep fraction and normalized for total L1 protein concentration, we observed a dramatic defect in DNA encapsidation efficiency of C428S and C175S virions (Fig. 5A–B and Fig. 7C). In contrast to data in Fig. 5B, C185S virions contained equal or more viral genomes per total L1 protein concentration compared to wild-type. The discrepancy between the DNA encapsidation efficiencies versus total L1 protein concentration might be due to extensive capsid instability of C185S capsids that do not contain a viral genome. In this scenario, only C185S capsids that have properly encapsidated and protected their genomes from endonucleases migrate into the highly infectious fractions. Another possibility would be that C185S virions are much smaller than wild-type virions, but still contain a full-length genome. These possibilities are supported by previous data utilizing HPV16 VLPs, which depict C185S capsids as smaller than wild-type capsids, and C175S capsids as much larger than wild-type capsids [16].

### Oxidative glutathione (GSSG)-induced treatment of wild-type and mutant virions

**Figure 9:**
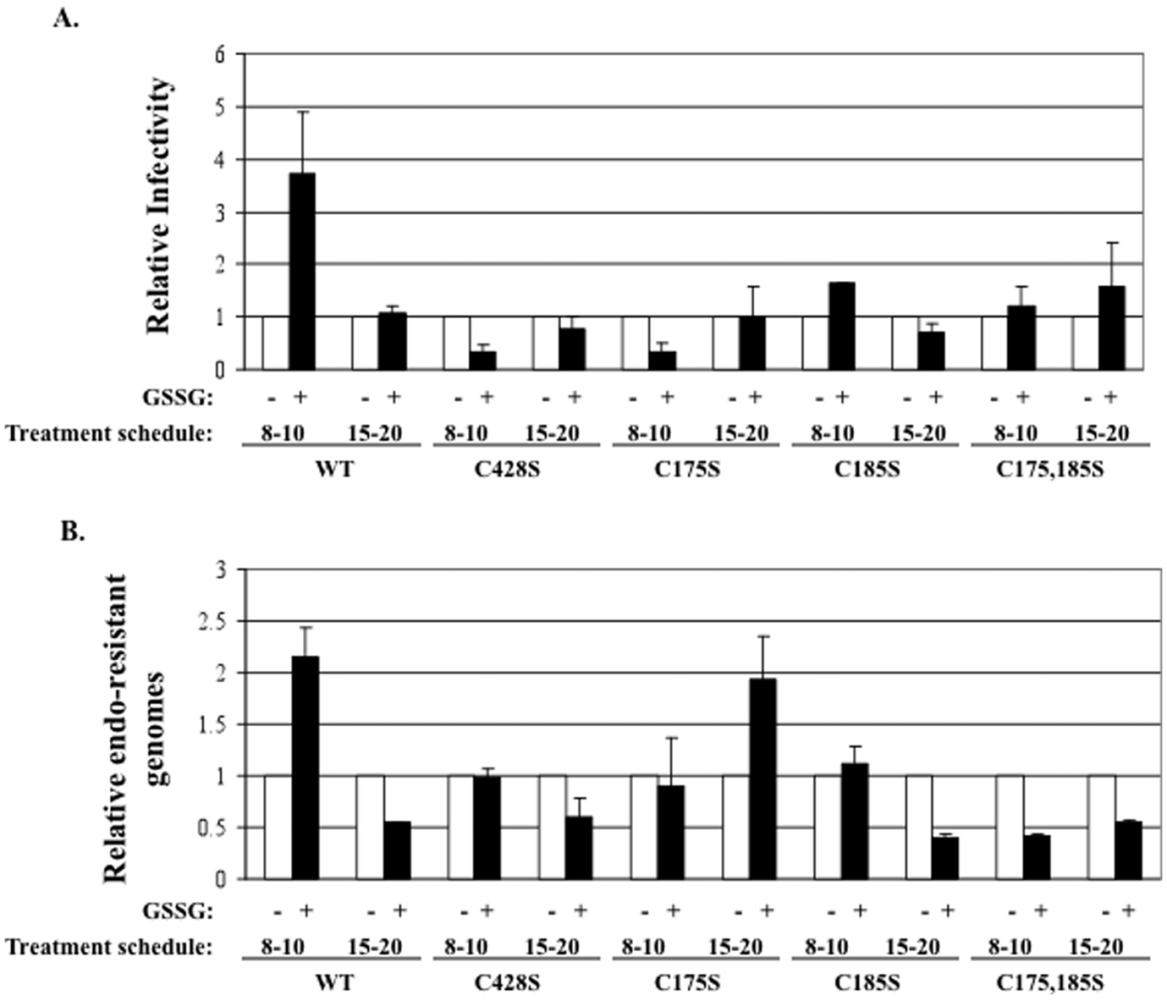
GSSG treatment alters viral titers and infectivity of tissues. (A) Relative RT-qPCR infectivity assay of wild-type (WT), C428S, C175S, C185S, and C175,185S 10 and 20-day viruses made from untreated (white) versus 5 mM GSSG-treated (black) organotypic cultures treated during days 8 through 10 or during days 15 through 20. Untreated 10-day WT was set to 1.0. (B) SYBR green-based DNA encapsidation assay of wild-type (WT), C428S, C175S, C185S, C175,185S 10 and 20-day viruses made from untreated (white) versus 5 mM GSSG-treated (black) organotypic cultures treated during days 8 through 10 or during days 15 through 20. Untreated 10-day WT was set to 1.0. Results are means and standard errors for three independent experiments.

Previous studies have shown that treatment of HPV PsV or HPV-infected organotypic raft cultures with 5 mM GSSG enhances capsid maturation. However, work with PsV suggests that GSSG treatment also results in an increase in misfolded capsids since they are more susceptible to partial tryptic proteolysis than untreated capsids [6], [23]. Our previous research has shown that treatment of HPV16-infected organotypic raft cultures with 5 mM GSSG starting on day 8 and ending on day 10, followed by harvesting of the tissue, leads to enhanced infectivity [23]. Similar results have also been observed through the addition of low concentrations of the mildly oxidizing agent DMSO (data not shown). Since previous research utilizing HPV PsV showed a role for C428S and C175S in capsid maturation, we tested whether or not C428S, C175S, C185S, or C175,185S mutant viruses would respond to GSSG-treatment as compared to wild-type virus (Fig. 9A–B) [6]. Early treatment of wild-type HPV16-infected organotypic tissues resulted in an approximately 3.5-fold increase in infectivity and a 2-fold increase in total endonuclease-resistant genomes (Fig. 9A–B). As previously reported, this phenomenon only occurred when treated with GSSG starting on day 8 and ending on day 10 (Fig. 9A–B) [23]. It did not occur when treated with GSSG starting on day 15 and ending on day 20 (Fig. 9A–B). None of the mutant viruses C428S, C175S, C185S, or C175,185S responded to GSSG-treatment when starting on day 8 and ending on day 10 (Fig. 9A–B). In fact, similar to data in Fig. 5C which showed a decrease in titer of 20-day mutant viruses compared to 10-day mutant viruses, titers and infectivity appeared to decrease in most instances upon treatment with 5 mM GSSG (Fig. 9A–B). These results suggest that under the influence of the natural tissue-spanning redox gradient between days 10 and 20, or via early treatment of virions with 5 mM GSSG, mutant virions appear to be destabilized either by a lack of stabilizing disulfide bonds or through incorrectly formed intra- and/or interpentameric disulfide bonds between the mutant L1 proteins.

## Discussion

In this study, we sought to determine individual temporal roles for the highly conserved HPV16 L1 cysteine residues: C428, C175, and C185 in capsid assembly within the context of stratifying and differentiating human epithelial tissue, HPV's natural host environment. Previous studies have depicted that C428S and C427S mutations in HPV16 and HPV33 VLPs, respectively, lead to the complete loss of capsid integrity, being disrupted into their respective capsomeres [16], [18], [20]. These studies have been extended to HPV16 PsV whereby C428S mutations also lead to the failure of capsids to form mature particles, suggesting a role for this residue in initial assembly and perhaps maturation [6]. A role for C428 in the formation of a critical interpentameric disulfide bond is supported by biochemical and cryo-electron microscopy data of non-reduced L1 dimers from HPV18 VLPs whereby an interpentameric disulfide bond between C175 and C429 is formed [14], [21]. A recent high-resolution cryo-electron microscopy image of native BPV also confirms the presence of a C171–C426 interpentameric disulfide bond [21]. However, the C175S substitution in both HPV16 VLPs and PsV does not lead to reciprocal complete disruption of capsids to capsomeres as is seen with the C428S mutant in VLPs and PsV. Instead, long tubular structures are formed as seen via EM and aberrant dimer formation is observed via Western blot analysis [6], [16]. These results suggest that, in HPV16, interpentameric contacts are possible without C175.

Thought to reside on an external loop neighboring C175, it has been suggested by others that C185 might form similar interpentameric contacts with C428 with only minor structural rearrangements of this loop [6], [13], [16], [21]. While the most recent high-resolution cryo-electron microscopy image reconstruction of native BPV virions only depicted a disulfide bond homologous to HPV16's C175–C428 disulfide bond and not a C185–C428 disulfide bond, this might be due to the low abundance or asymmetry of C185–C428 disulfides which are averaged out of the reconstruction [21]. Our data suggests that C175–C428 may occur prior to C185 and that when C175 is missing the C185–C428 bond can occur. It is possible that C185–C428 occurs early and may assist in preparing for a switch to C175–C428. Additionally, the double mutation, C175S/C185S prevents the deleterious C428-C185 bond to form/remain in the oxidative environment of 20 day tissues in the absence of C175.

Our data suggests that all three conserved cysteine residues, C428, C175, and C185 are critical for proper assembly and maturation of native HPV16 virions. While no individual substitution led to the complete disruption of particles as was previously seen with C428S mutations in HPV16 VLPs and PsV, each substitution undermined particle formation efficiency and stability. These virions were more susceptible to intrusion of endonucleases into the capsid lumen, and less able to withstand the forces of ultracentrifugation [6], [16], [18]. In addition, all three mutations led to a deficiency in capsid maturation when exposed either to the natural, tissue-spanning redox gradient or through artificial treatment with GSSG. Further, studies of HPV16 VLP assembly showed rapid assembly under acidic conditions (pH 5.2) indicative of a kinetic trap due to overinitiated assembly [33]. Only at pH 6.2, 7.2, and 8.2 did particles avoid aggregation and particles produced at pH 7.2 disassembled slower than the rest. We hypothesize that alterations in the chemical environment of tissue can affect the quality and quantity of the resulting papillomavirus particle [33].

Contrary to work with VLPs and PsV, in the context of organotypic culture-derived native virions, the C428S mutation led to approximately 50% of wild-type titers within 10-day viral preparations suggesting that hydrophobic interactions and/or cellular factors are involved in reinforcing the integrity of C428S virions [6], [16], [18], [34], [35], [36]. We hypothsize that within the suprabasal compartment of 10-day tissues, assembly of wild-type virions is promoted by extensive hydrophobic interactions. In this temporospatial environment, these interactions most likely take place between neighboring L1 pentamers, and between L1 pentamers and monomeric L2, assisted by cellular chaperones [7], [13], [14], [17], [21], [34], [35], [36], [37], [38]. Knowledge of the structure, location, and number of L2 molecules in the capsid is at best sparse. While effects on L2 are possible reasons for the phenotypes observed too little is known about L2 in the capsid to draw any conclusions.

In Fig. 5A–C, it is apparent that the C175S mutation severely limits the number of endonuclease-resistant genomes that are present within 10-day virus, while C185S and C175,185 mutations do not appear to be limiting. We hypothesize that it is possible for a capsid to contain a mixture of both C175–C428 and C185–C428 disulfide bonds with C185–C428 disulfide bonds being in the minority. In this scenario, the substitution of C175 for serine inhibits the formation of C175–C428 disulfide bonds, and allows for the formation of a larger percentage of C185–C428 interpentameric disulfide bonds that are less efficient in stabilizing the particle. Another possibility is that C175S mutations might alter an unknown early role for C175 in assembly. However, the formation of improper C185–C428 disulfides is supported by previous studies utilizing C175S mutant HPV16 VLPs and PsV which lead to the production of abnormal tubular structures via EM and the production of aberrant L1 dimers, respectively [6], [16]. But nonreducing Western blot analysis of 10-day C175S virus does not depict L1 dimer formation, suggesting that if C185-C428 disulfides do take place, they are in the minority (Fig. 4A).

Analysis of 20-day viruses reveals the temporal importance of C175, C428 and C185. Titers remain low in 20-day C175S viral preparations as they had in 10-day viral preparations. Titers for C428S and C185S mutations were significantly down within 20-day viral preparations (Fig. 5B). This observation suggests that either, (i) C185 and C428 form a disulfide during maturation within tissue, (ii) that C185 effects a more extensive C175–C428 disulfide bonds, (iii) that C185S mutations alter the environment within the E–F loop >which makes it difficult for C175–C428 disulfides to form, or (iv) the potential structural effect of the serine residues could induce a shift in the loops. Due to previous structural analyses of capsids which have depicted a C175–C428 endpoint disulfide bond, we hypothesize that a transient C185–C428 disulfide bond occurs which primes final C175–C428 disulfide bonds [14], [21]. This hypothesis is in line with previously reported studies [6], [16].

Further supporting the role of a complex interplay of disulfide bonding between C428, C175, and C185, is our finding that C428S, C175S, C185S, and C175,185S 20-day viral preparations have a significant decrease in titers compared to their 10-day counterparts. This suggests that each of these residues is important in the efficient accumulation of mature capsids (Fig. 5C). This result is supported by GSSG treatment of wild-type and mutant-infected tissues whereby virions within the mutant-infected tissues failed to respond to the oxidizing agent (Fig. 9). When normalized by titer or L1 protein levels the particles that do form appear to be less infectious. This could be due to the overall structure of the formed particles is not correct, thus an indirect effect on attachment, entry, or trafficking.

## Materials and Methods

### DNA reagents

pBSHPV16(114/B) DNA, a generous gift from M. Dürst (Kirnbauer et al., 1993) was utilized as the template for site-directed mutagenesis using Strategene's Quikchange II XL Site-Directed Mutagenesis Kit. Following transformation and selection of the mutant amplimers into XL10 Ultracompetent *E. coli* cells (Stratagene, La Jolla, Calif.), we controlled for polymerase fidelity and/or random mutations that may arise during PCR amplification by extracting plasmid DNA from many isolated bacterial clones and sequencing the L1 ORF of each clone to verify the correct incorporation of nucleotide substitutions and the absence of spurious mutations elsewhere in the L1 ORF. Multiple mutant viral genomic clones containing correctly mutagenized sequences were isolated per each mutation and utilized in subsequent experimentation. To create a full-length, HPV16(114/B) genome with a Cys175Ser substitution, the two complimentary oligonucleotides: forward 5′CTGGGGCAAAGGATCCCCATCTACCAATGTTGCAGTAAATC3′ and reverse 5′GATTTACTGCAACATTGGTAGATGGGGATCCTTTGCCCCAG3′, were used with the change of G to C at nucleotide 6161. To create a full-length, HPV16(114/B) genome with a Cys185Ser substitution, the two complimentary oligonucleotides: forward 5′GCAGTAAATCCAGGTGATTCTCCACCATTAGAG3′ and reverse 5′CTCTAATGGTGGAGATCACCTGGATTTACTGC3′, were used with the change of G to C at nucleotide 6191. To create a full-length, HPV16(114/B) genome with a Cys428Ser substitution, the two complimentary oligonucleotides: forward 5′GTAACCCAGGCAATTGCTTCTCAAAAACATACACCTCC3′ and reverse 5′GGAGGTGTATGTTTTTGAGAAGCAATTGCCTGGGTTAC3′, were used with the change of G to C at nucleotide 6917. To create a full-length, HPV16(114/B) genome with Cys175,185Ser substitutions, the two complimentary oligonucleotides: forward 5′GCAGTAAATCCAGGTGATTCTCCACCATTAGAG3′ and reverse 5′CTCTAATGGTGGAGATCACCTGGATTTACTGC3′, were used with the change of G to C at nucleotides 6161 and 6191, respectively. The Cys175,185Ser amplimers were made utilizing the Cys175Ser template. In all instances, the most prevalent serine codon in the HPV16(114/B) L1 ORF, TCT, was utilized.

### Differentiation-dependent native virion production

Immortalized HFK lines which stably maintained episomal HPV16 DNA were grown in monolayer culture using E medium in the presence of mitomycin C-treated J2 3T3 feeder cells [39]. Raft tissues were grown as previously described [27], [39], [40]. For oxidized glutathione (GSSG) (Sigma) treatment, E-media was supplemented with the appropriate concentration and fed to organotypic cultures either beginning on day eight followed by tissue harvest on day ten (8 to 10-day), or beginning on day fifteen followed by tissue harvest on day twenty (15 to 20-day).

### Keratinocyte cultures, and electroporation

Primary human foreskin keratinocytes (HFKs) were isolated and grown from newborn circumcision as described previously [27]. For electroporations, 30 µg of wild-type or mutagenized pBSHPV16(114/B) DNA was digested with *BamHI*, linearizing the viral DNA at nucleotide 6151 in L1 and separating it from the vector sequence. HFKs were electroporated with the prepared DNA as described previously [27], [39]. Multiple cell lines were obtained for each wild-type and mutant construct.

### Southern blot hybridization

Total cellular DNA was isolated as previously described [39]. Briefly, 5 µg of total cellular DNA was digested with *BamHI*, which linearizes the HPV16 genome. Samples were separated by 0.8% agarose gel electrophoresis and transferred onto a GeneScreen Plus membrane (New England Nuclear Research Products) as previously described [41]. Hybridization of the membrane utilized an HPV16-specific, whole genomic probe as previously described. [39].

### Histology and immunohistochemical staining

Tissues were fixed in 10% neutral buffered formalin, and embedded in paraffin. Four-micrometer sections were cut and stained with hematoxylin and eosin as previously described [39].

### HPV isolation

For Optiprep fractionation, RT-PCR, RT-qPCR, and qPCR-based DNA encapsidation assays, 3-raft tissues viral preparation or VLP and PsV lysates were prepared by dounce homogenization in 500 µl phosphate buffer (0.05 M Na-phosphate [pH 8.0], 2 mM MgCl_2_). Homogenizers were rinsed with 250 µl phosphate buffer. 1.5 µl (375 units) Benzonase (Sigma) was added to 750 µl of CVPs and incubated at 37°C for 1 hour. Samples were brought to 1 M NaCl by adding 130 µl ice cold 5 M NaCL. Then, samples were vortexed and centrifuged at 4°C for 10 minutes at 10,500 rpm in a microcentrifuge. Supernatants were stored at −20°C.

### Optiprep purification of virions

Optiprep purification was performed as described previously [12], [42]. Briefly, 27%, 33%, 39% Optiprep gradients were produced by underlayering. Gradients were allowed to diffuse for 1 to 2 h at room temperature. Then, 600 µl of clarified benzonase-treated virus preps were layered on top of the gradient. Tubes were then centrifuged in a SW55 rotor (Beckman) at 234,000× *g* for 3.5 h at 16°C. After centrifugation, 11–500 µl fractions were collected from the top of each tube.

### Quantitative RT-qPCR infectivity assays

HaCaT cells were grown to confluence in Dulbecco's modified Eagle's medium supplemented with 10% fetal bovine serum, 2 mM glutamine, 1 mM pyruvate, 100 units/ml penicillin, and 100 µg/ml streptomycin and seeded 50,000 cells/well in 24-well plates. 50 µl of HPV16 viral stock was diluted with cell culture medium to a total volume of 0.5 ml. Medium was aspirated from HaCaT cells and 0.5 ml of diluted virus was added per well. One well on each plate received 0.5 ml of medium without virus as a negative control. The cells were incubated with the virus for 48 h at 37°C. mRNA was harvested with the SurePrep TrueTotal RNA Purification Kit (Fisher Scientific). DNA contamination of columns was insignificant in that the optional on-column DNase-I treatment of extracted mRNA had no effect on downstream signal. Amplification of both the viral target and endogenous cellular control target was performed using a duplex format in 0.2 ml, 96-well PCR plates (BIO-RAD) with a total reaction volume of 25 µl. All reactions containing RNAs from virus-infected cells were performed in duplicate or triplicate. Reverse transcription and quantitative PCR were performed in the same closed tube with approximately 250 ng of total RNA per reaction using the Quantitect Probe RT-PCR Kit (Qiagen). HPV16 E1∧E4 primers used were the splice-site straddling, forward 5′GCTGATCCTGCAAGCAACGAAGTATC3′ (nt 868–3372) and reverse 5′GGATTGGAGCACTGTCCACTGAG 3′ (nt 3535–3557) at final concentrations of 4 µM. A fluorogenic, dual-labeled, HPV16 E1∧E4 probe of 5′ 6-FAMCACCGGAAACCCCTGCCACACCACTAAGBHQ-1 3′ (nt 3493–3520) was utilized at a final concentration of 0.2 µM to detect E1∧E4 cDNA. Primers and probe were developed using Gene Link Software: OligoAnalyzer 1.2, and OligoExplorer 1.2. TATA-binding protein (TBP) amplicons were created using primers 5′ CACGGCACTGATTTTCAGTTCT 3′ (nt 627–648) and 5′ TTCTTGCTGCCAGTCTGGACT 3′ (nt 706-686) at final concentrations of 0.125 µM. TBP amplicons were detected by the fluorogenic TaqManTM probe 5′HEX TGTGCACAGGAGCCAAGAGTGAAGA BHQ-13′ used at 0.2 µM. TBP primer sequences were obtained from those previously described [43]. All primers were synthesized by Integrated DNA Technologies (Coralville, IA). All QRT-PCR reactions were performed using the iQ5 (BIO-RAD). Cycling conditions were 50°C for 30 min (reverse transcription) and 95°C for 15 min, followed by 42 cycles of 94°C for 15 s and 54.5°C for 1 min. Amplification efficiencies of each primer set was 93% for E1∧E4 and 97% for TBP. Relative quantities of viral target cDNA were determined using REST© software.

### qPCR-based DNA encapsidation assay

To detect endonuclease-resistant genomes in viral preparations or Optiprep fractions, only benzonase-treated samples were used so that all non-encapsidated genomes were digested. To release all encapsidated viral genomes, 10 µl sonicated virus preparation or 20 µl Optiprep fraction was added to 2 µl Proteinase K, 10 µl 10% SDS, 2 µl pCMV-GFP (140 ng/µl) carrier DNA, and brought up to 200 µl with Hirt buffer. Tubes were rotated at 37°C for 2 hours. Immediately, an equal amount of phenol-chloroform-isoamyl alcohol (25∶24∶1) was added and the mixture was extracted for the aqueous phase. An equal amount of chloroform was added and again extracted for the aqueous phase. DNA was EtOH precipitated overnight at −20°C. After centrifugation, the DNA pellet was washed with 70% EtOH and resuspended in 20 µl TE overnight. To detect viral genomes or cellular DNA, a Qiagen Quantitect SYBR Green PCR kit was utilized. Amplification of the viral target was performed in 0.2 ml, 96-well PCR plates (BIO-RAD) with a total reaction volume of 25 µl. l µl of each endonuclease-resistant viral genome prep was analyzed in triplicate for each independent experiment. Amplification of HPV16 genomes was performed using 0.3 µM 5′CCATATAGACTATTGGAAACACATGCGCC3′ as the forward primer (nt 2839–2868) and 0.3 µM 5′CGTTAGTTGCAGTTCAATTGCTTGTAATGC3′ as the reverse primer (nt 2960–2989). Amplification of human repetitive Alu DNA was performed using 0.3 µM 5′GTCAGGAGATCGAGACCATCCC3′ as the forward primer and 0.3 µM 5′TCCTGCCTCAGCCTCCCAAG3′ as the reverse primer [44]. Oligonucleotides were synthesized by Integrated DNA Technologies (Coralville, IA). A standard curve was generated by amplifying serially-diluted pBSHPV16 copy number controls. Acceptable R^2^ values for standard curves were at or above 0.99. A Bio-Rad iQ5 Multicolor Real-Time qPCR machine and software were utilized for PCR amplifications and subsequent data analysis. The PCR thermocycling profile was as follows: 15 min. hot-start at 95°C, followed by 40 cycles at 15 sec. at 94°C, 30 sec. at 52°C, and 30 sec. at 72°C. Data analysis commenced during the extension phase.

### Immunoblot analysis

40 µl aliquots from Optiprep fractions were boiled for 10 minutes in 6% 2-ME loading buffer and loaded on 8% polyacrylamide gels. To detect HPV16 L1, anti-HPV16 L1 monoclonal antibody (Camvir-1; BD Pharmingen) was utilized at a dilution of 1∶4,000 per manufacturer's recommendations. For nonreducing gels, loading buffer without 2-ME was utilized. Samples were boiled for 10 minutes at 70°C and loaded on 6% polyacrylamide gels. L1 was detected using the anti-HPV16 L1 monoclonal antibody, H16.J4 [45].
